# The Role of Optineurin in Antiviral Type I Interferon Production

**DOI:** 10.3389/fimmu.2018.00853

**Published:** 2018-04-26

**Authors:** Ahmed Outlioua, Marie Pourcelot, Damien Arnoult

**Affiliations:** ^1^INSERM, UMR_S 1197, Hôpital Paul Brousse, Villejuif, France; ^2^Université Paris-Saclay, Paris, France; ^3^Molecular Genetics and Immunophysiopathology Research Team, Health and Environment Laboratory, Aïn Chock Faculty of Sciences, Hassan II University of Casablanca, Casablanca, Morocco; ^4^ANSES, INRA, ENVA, UPEC, UMR_1161 Virology, LabEx IBEID, Maisons-Alfort, France

**Keywords:** TBK1, optineurin, Golgi apparatus, viruses, innate immunity

## Abstract

After a viral infection and the stimulation of some pattern-recognition receptors as the toll-like receptor 3 in the endosomes or the RIG-I-like receptors in the cytosol, activation of the IKK-related kinase TBK1 leads to the production of type I interferons (IFNs) after phosphorylation of the transcription factors IRF3 and IRF7. Recent findings indicate an involvement of K63-linked polyubiquitination and of the Golgi-localized protein optineurin (OPTN) in the activation of this crucial kinase involved in innate antiviral immunity. This review summarizes the sensing of viruses and the signaling leading to type I IFN production following TBK1 activation through its ubiquitination and the sensing of ubiquitin chains by OPTN at the Golgi apparatus.

After a viral infection, the innate immune response is the first line of defense. Replication of a virus into host cells engenders molecular marks that are called pathogen-associated molecular patterns (PAMPs). The presence of PAMPs like viral nucleic acids is sensed by germline-encoded pattern-recognition receptors (PRRs) that generate a series of signaling pathways conducting to the prompt production of pro-inflammatory cytokines and type-I interferons (IFNs) (IFNα/IFNβ) (1, 2) to establish the antiviral immune response.

## Type I IFNs

Type I IFNs include 5 different classes, expressed in humans on the chromosome 9 locus: IFN-α (itself divided into 13 subtypes), -β, -ω, -ε, and -κ. They form a large family of cytokines that regulate the early development of viral infections (3, 4). Most cell types following infection produce IFN-β, whereas hematopoietic cells, particularly plasmacytoid dendritic cells, are dedicated in the secretion of IFN-α. On the other hand, the IFN-ε and -κ are not induced following the stimulation of PRRs. However, they are constitutively expressed by the epithelial cells of the female genital tract and by the keratinocytes, respectively (5, 6). IFN-κ has only a weak antiviral activity, IFN-ε contributes toward the protection against sexually transmitted infections by HSV-2 and *Chlamydia muridarum*.

Classical type I (α and β) IFNs exert their function *via* heterodimeric receptors consisting of two subunits: IFNAR1 and IFNAR2. Activation of these receptors by IFNs induces the JAKs/STATs signaling pathway. Once activated by phosphorylation, the STAT1 and STAT2 transcription factors form a heterodimer that associates with the IRF9 factor to form a tripartite complex called ISGF3. This complex moves into the nucleus where it binds to the ISRE sequence present in the transcription promoters of a series of genes called IFN-stimulated genes (ISGs). The ISGs inhibit the viral dissemination and stimulate the adaptive immune response (7). The lack of type-I IFN signaling conducts to a serious immunodeficiency with a high sensitivity to viral infection (8).

## The Sensing of Viruses and the Signaling Leading to Type I IFN Induction

**Figure 1:**
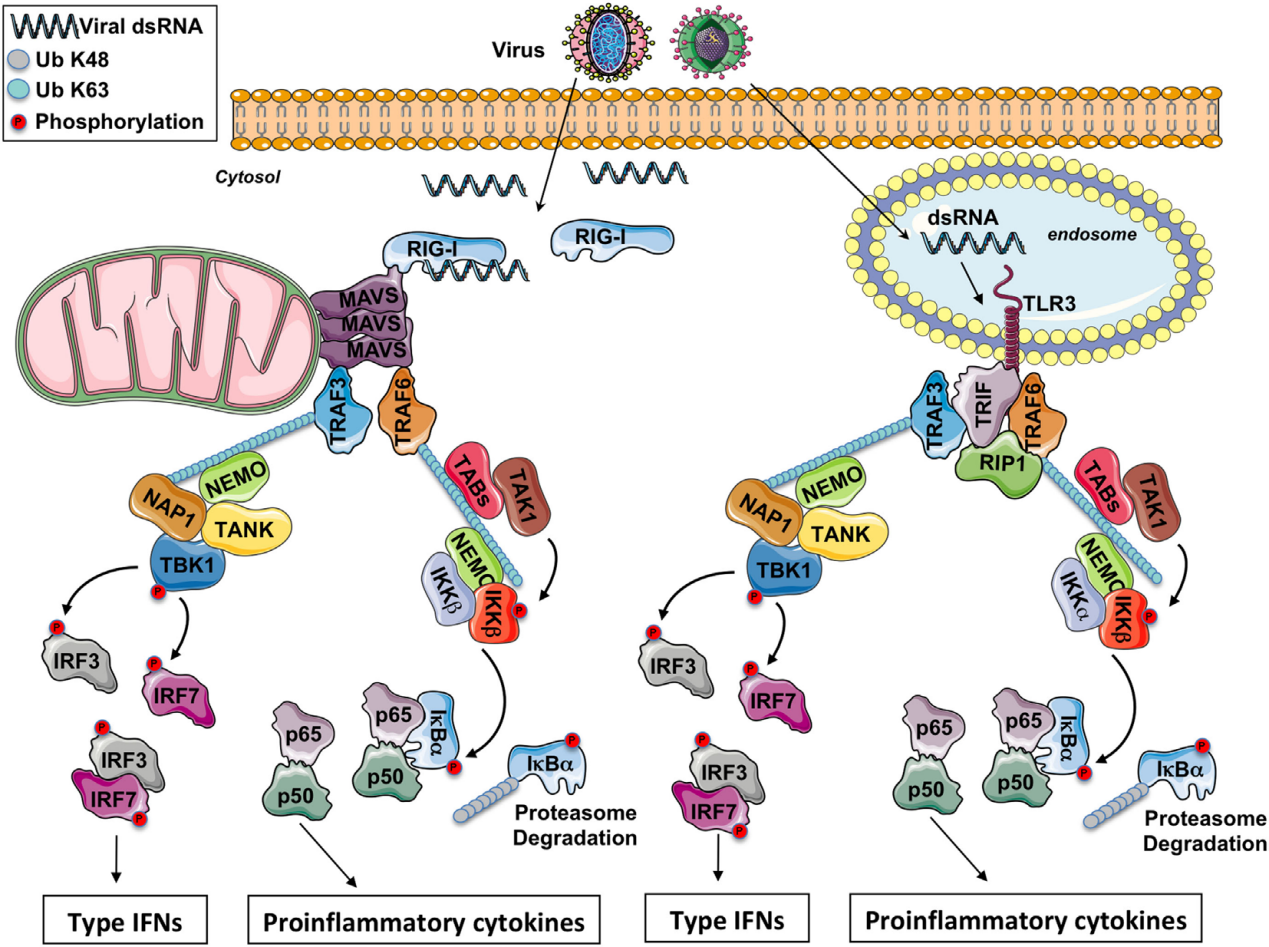
The RIG-I-like receptors and toll-like receptor (TLR) 3 signaling pathway. After a viral infection, double-strand RNA (dsRNA) in the cytosol is sensed by RIG-I while it is sensed by TLR3 in the endosomes. Through the adaptors MAVS and TRIF, respectively, some E3 ubiquitin ligases TRAFs are recruited and polyubiquitinated. On the K63-linked ubiquitin chains, on the one hand, the IKK complex is activated leading to NF-κB activation and the production of pro-inflammatory cytokines and on the other hand, the IKK-related kinase TBK1 is activated triggering the phosphorylation of IRF3 and IFR7 for the production of type I IFNs.

The virus detection involves various classes of PRRs including the endosomal toll-like receptors (TLRs), the cytosolic retinoic acid-inducible gene-I (RIG-I)-like receptors (RLRs), and the cytosolic DNA receptors (1, 2). In this review, we only focus on viruses with double-strand RNA (dsRNA). All antiviral PRRs generate type-I IFN expression, but the signaling modules involved are different between PRRs. Nonetheless, the recruitment of adaptor proteins to establish a platform with the cellular ubiquitin ligases TRAFs is one common feature of all these signaling pathways. For instance, in endosomes, viral dsRNA is detected by TLR3, while in the cytosol viral RNA is sensed by RLRs, allowing the recruitment of the adaptors TRIF and MAVS (this protein being anchored into the mitochondrial outer membrane), respectively (Figure 1).

Toll-like receptor 3 detects viral dsRNA in the endolysosome, but TLR3 can also detect poly I:C, a synthetic analog of dsRNA. After stimulation, TLR3 recruits the adaptor protein TRIF. Through TRAF-binding motifs localized in its N-terminal portion, TRIF associates with the cellular ubiquitin ligases TRAF3 and TRAF6. Interestingly, in its C-terminal domain, TRIF contains an RIP homotypic interaction motif required for its interaction with RIPK1 and RIPK3 to promote necroptosis in some conditions (9). TRADD, an essential adaptor for TNFR signaling, has been initially reported to be required in the TRIF-dependent signaling pathway (10, 11). In complex with TRADD and FADD, RIPK1 is ubiquitinated, an event required for NF-κB activation. Moreover, in response to poly I:C, caspase-8 or caspase-10 is activated by FADD, and the cleaved form of caspases activates NF-κB (1). Nevertheless, recent reports have revealed that the necessity for caspase-8 (and likely also FADD) in immune cell proliferation is explained by the suppression of RIPK3 that triggers necrosis, so that the contribution of caspase-8, FADD, TRADD, and RIPK1 in the TRIF-mediated signaling is therefore questioned (12). Hence, RIPK1 and TRAF6 ubiquitination is likely more required for NF-κB activation. On the K63-linked poly-ubiquitin chains, after activation by TAK1, the IKK complex (composed by the subunits IKKα, IKKβ, and NEMO) triggers the phosphorylation then the K48-linked poly-ubiquitination of the inhibitor IκBα and its subsequent proteasomal degradation, occasioning the liberation of free NF-κB dimers and the production of pro-inflammatory cytokines.

TRAF3 is critical for activating TBK1, an IKK-related kinase (13, 14). TRAF3 acts as an E3 ubiquitin ligase and undergoes K63-linked auto-ubiquitination in response to TLR3 stimulation. TBK1 or its close homolog IKK-ε phosphorylates IRF3 and IRF7, then IRF3 and IRF7 dimers translocate into the nucleus, leading to the production of type I IFNs and ensuing expression of IFN-inducible genes. Finally, various proteins modulate TBK1 activation. It has been reported that TBK1 interacts with TANK, NAP1, and SINTBAD (which is similar to NAP1) (15–17). These molecules show similarities in their coiled-coil domains and importantly contain a TBK1-binding motif, but the connection between these molecules in the TRIF signaling has not been fully identified yet.

Toll-like receptor 3, like other TLRs, is almost totally localized in intracellular compartments as endosomes (18) so that the viral RNA present in viral genomes already liberated into the cells or synthetized in the cytosol is not accessible to TLR3. Nevertheless, RNA viruses can still be detected in the cytoplasm through the RLRs. Three molecules compose this family of cytosolic PRRs: RIG-I, MDA-5, and LGP2. LGP2 is currently supposed to function as a regulator of RIG-I and MDA5 signaling (19) while RIG-I and MDA-5 are both prototypical PRRs. RIG-I and MDA-5 have dsRNA, 5′-tri or -diphosphate RNA as ligand (19, 20). Reducing the length of dsRNA changes MDA5 ligand to an RIG-I ligand (21). RIG-I and MDA-5 are cytosolic helicases with ATPase activity containing a C-terminal regulatory domain required for the binding to viral RNA and two tandem CARD domains in the N-terminus (19). The CARDs of RIG-I are released from the regulatory domain repression after recognition of specific panhandle RNA structures and K63-linked poly-ubiquitination by the E3 ubiquitin ligases TRIM25 and RIPLET (19). This conformational change allows a presumed interaction between the two CARD domains of RIG-I or MDA-5 with the CARD domain of MAVS, a protein anchored into the mitochondrial outer membrane through a transmembrane domain in its C-terminal region (22). *In vivo* studies with MAVS deficient mice have demonstrated an essential role of MAVS in antiviral innate immunity (23).

In addition to its interaction with RIG-I or MDA5, the CARD domain of MAVS is involved in the foundation of functional prion-like aggregates required for the production of type I IFNs (24). MAVS was reported to associate with TRAF3 (25). In complex with NEMO (26), TANK (15), and NAP1 (17), TRAF3 controls the activity of TBK1 which phosphorylates the transcription factors IRF3 and IRF7. NEMO has been proposed to function as a sensor of K63 poly-ubiquitin chains to activate TBK1 (27). MAVS polymers also recruit TRAF6 (28), and this E3 ubiquitin ligase makes K63-linked poly-ubiquitin chains on which is recruited and activated the IKK complex, next triggering NF-κB activation.

The signaling downstream of TLR3 and RLRs piloting the production of type I IFNs and of pro-inflammatory cytokines is depicted in Figure 1.

## TBK1, a Key Kinase in Type I IFN Production

In addition to IKKα and IKKβ, the IKK kinase family contains two non-canonical family members: TBK1 and IKKε (also called IKK*i*) (29). The canonical and non-canonical IKK kinases share about 30% sequence identity. While TBK1 and IKKε have comparable biochemical properties *in vitro*, they have really different functions *in vivo* (30–33). As described earlier, TBK1 is a pivotal regulator of type I IFN production. Indeed, *in vivo* the lack of TBK1 has a strong effect on type I IFN induction after viral infection as it phosphorylates IRF3 and IRF7 (30–32). IKKε is only required for the activation of IFN-stimulated genes, not for type-I IFN expression. IKKε phosphorylates a specific serine residue on the transcription factor STAT1, regulating therefore the formation of IFN-inducible transcription factor complexes (33).

Between the N-terminal kinase domain and the C-terminal dimerization/scaffolding domain, TBK1 contains a predicted ubiquitin-like domain (ULD) that is present in the different IKK kinases. In TBK1 or IKKε, kinase activation and substrate phosphorylation in cells are severely impaired after deletion or mutation of the ULD (34, 35).

As mentioned earlier, TBK1 is a pivotal player in the production of type-I IFNs as the genetic ablation of TBK1 has a strong influence on type-I IFN induction after viral infection (30–32, 36). Unlike IKKε, TBK1 is constitutively expressed, and TBK1-deficient mice exhibit embryonic lethality due to a general apoptosis in the liver, a phenotype that closely resembles IKKβ-deficient mice (37, 38). Phosphorylation on the serine 172 within the classical kinase activation loop regulates TBK1 activation. Results from genetic or pharmacological inhibition experiments suggest that TBK1 can be activated by IKKβ, but it is more likely activated by *trans*-autophosphorylation (39, 40). Importantly, after viral infections, K63-linked poly-ubiquitination of TBK1 lysine residues as additional post translational modifications are required for type I IFN production (41, 42). The mechanisms of TBK1 activation are not clearly understood. However, it has been proposed that TBK1 *trans-*autophosphorylation relies on the subcellular localization of TBK1 *via* different adaptor proteins that guide TBK1 to distinct signaling complexes for different cellular responses (34, 40, 43, 44).

## TBK1 Ubiquitination is Required for Activation

Ubiquitination plays a central role in the transmission of signals necessary for the activation of antiviral innate responses. Proteins, targeted in particular by K63-linked poly-ubiquitination, are subsequently capable of engaging new interactions with proteins containing ubiquitin-binding domains (UBDs), thus contributing to the formation of functional signalosomes. Several proteins like RIG-I, MAVS, TRAF3, or TANK are subject to K63-linked ubiquitination, when activating signaling pathways are initiated downstream of TLR3 and RLRs. Recently, it has been demonstrated that K63-linked poly-ubiquitination plays an essential role in TBK1 activation (41, 42).

In a context of cellular transformation and immune signaling, Zhou et al. have investigated the essential role of K63-linked poly-ubiquitination in the regulation of IKKε (45). Interestingly, the two essential residues for poly-ubiquitination of IKKε, Lys30, and Lys401 are conserved in its counterpart TBK1. It has been further demonstrated that the ubiquitination of TBK1 on Lys30 and Lys401 is also essential for its activity (42). Structurally, these two residues are on opposite sides of the monomers but are in the vicinity as part of the catalytically active dimer of TBK1 (42).

Finally, it has been proposed that the E3 ubiquitin ligases Mind Bomb (MIB1 and MIB2) and NRDP1 are involved in the K63 ubiquitination of TBK1 to allow its activation (41, 46, 47). However, no animal models and *in vivo* experiments have confirmed the role of MIBs and NRPDP1 as key E3 ubiquitin ligases involved in innate immunity through TBK1 ubiquitination and ensuing activation. Hence, further studies are absolutely required to validate the role of these E3 ligases in TBK1 activation or to discover new E3 ligases involved in this process. This is really a gap in the field as it is now accepted that K63 ubiquitination of TBK1 is required for activation of this kinase.

## TBK1 Activation at the Golgi Apparatus

Viral RNAs in endosomes are sensed by TLR3 while RNAs in the cytosol are detected by RLRs (2). Stimulation of these PRRs triggers the activation of TBK1 that plays an essential role in innate antiviral immunity through the phosphorylation of IRF3, a transcription factor crucial for the production of type I IFNs (30, 31, 36). Interestingly, some signaling pathways lead to TBK1 activation whereas the transcription factor IRF3 is not. Indeed, the stimulation of cells with the pro-inflammatory cytokines IL-1β or TNFα, the oncogenic transformation through the KRAS signaling or the induction of mitophagy, promotes the phosphorylation of TBK1 without any sign of IRF3 activation (48–51). To explain this, it has been suggested that, according to the cellular response, different adaptor proteins guide TBK1 to isolated signaling complexes, controlling therefore TBK1 *trans*-autoactivation and the substrate specificity, both being dependent on the subcellular distribution of TBK1 (34, 40, 43). Consistent with this hypothesis, after the stimulation of TLR3 or RLRs, it was recently described that the active (phosphorylated and ubiquitinated) form of TBK1 localizes at the Golgi apparatus and that the substrate IRF3 is phosphorylated (44). By contrast, no significant accumulation of p-TBK1^S172^ is observed at the Golgi apparatus after the cells are exposed to IL-1β or TNFα (unpublished observations), and in the case of mitophagy, active TBK1 is recruited to depolarized mitochondria (49). Thus, the localization of active TBK1 at the Golgi apparatus seems to be a condition for the phosphorylation of IRF3 by this kinase. IFR3 phosphorylation near the Golgi apparatus, which borders the nucleus where this transcription factor migrates after its activation, reduces the chances for IRF3 to meet some E3 ubiquitin ligases or phosphatases that are involved in the negative regulation of the signaling.

In response to TLR3 or RLRs stimulation, an elegant study has shown that TBK1 phosphorylates a conserved domain on the adaptor TRIF or MAVS, respectively (51). Once phosphorylated, the adaptor subsequently binds to IRF3 on a positively charged surface, hence allowing the recruitment of this transcription factor for its phosphorylation and activation by TBK1 (51). While active TBK1 localizes at the Golgi apparatus, this kinase has nevertheless the opportunity to phosphorylate the adaptors TRIF or MAVS to promote IRF3 recruitment and the resulting activation of this transcription factor. Indeed, the endosomes (where is recruited TRIF after TLR3 stimulation) through their trafficking and the mitochondrion (where is anchored MAVS) as a highly dynamic organelle, are always very close to the Golgi apparatus hence permitting active TBK1 to phosphorylate TRIF or MAVS, a process requisite for IRF3 activation (51). In agreement, p-TBK1^S172^ is detected at the mitochondria after activation of RLRs into HeLa cells following transfection of RIG-I CARDs, MAVS, or poly (I:C), or following infection of neuroepithelial stem cells with Zika virus (52, 53). Nevertheless, the detection of active TBK1 at the mitochondria or else may result from the release of molecular complexes formed at the Golgi apparatus where TBK1 had been initially activated (see below).

One of the major roles of the Golgi apparatus is related to protein secretion by exocytosis. It regulates vesicular trafficking and is the intermediate between the endoplasmic reticulum and the plasma membrane. Addressing and attachment of post-Golgi vesicles to the plasma membrane before vesicle fusion requires the involvement of a complex of eight proteins called the exocyst (also called translocon) (54). It should be pointed out that two studies have reported an important role of the exocyst and in particular of its Sec5 subunit (EXOC2) in the production of type I IFNs without, however, explaining how Sec5 could be involved (55, 56). Hence, at the Golgi apparatus, the active form of TBK1 phosphorylates IRF3 which after translocation into the nucleus induces the expression of type I IFNs and on the other hand, TBK1 may phosphorylate Sec5 to stimulate the secretion of type I IFNs. TBK1 would then have the capacity to induce both the synthesis and the secretion of type I IFNs.

## Optineurin (OPTN) Senses Ubiquitinated TBK1 for Its *Trans*-Autophosphorylation at the Golgi

The *trans*-autoactivation of TBK1 requires a K63-linked poly-ubiquitination on lysines 30 and 401 (41, 42). Recent findings suggest that the non-degradative ubiquitination of TBK1 allows this kinase to be targeted to the Golgi apparatus, a step required for its activation (44). TBK1 possesses more than 50 lysine residues conserved between humans and mice, so ubiquitination of specific lysine residues might influence the subcellular distribution of this kinase and the downstream signaling. For its targeting and activation at the mitochondrion for mitophagy (49, 50), it is currently not known whether TBK1 has to be ubiquitinated after the loss of mitochondrial transmembrane potential. Likewise, whether TBK1 is also ubiquitinated after cell exposure to TNFα or IL-1β remains to be determined.

Importantly, it was recently reported that at the Golgi apparatus, ubiquitinated TBK1 is sensed by OPTN causing its *trans*-autophosphorylation after TLR3 or RLR stimulation (44). OPTN, a protein localized in part at the Golgi apparatus (44, 57), has arisen as an important factor involved in the regulation of various physiological processes as protein secretion, cell division, membrane trafficking, mitophagy, and innate immunity (see the other reviews in this issue) (57). Of all the four NEMO-related ubiquitin-binding proteins (ABIN1, ABIN2, ABIN3, and OPTN), OPTN has the greatest sequence homology with NEMO, as it possesses the C-terminal zinc finger as well as the UBD (58).

**Figure 2:**
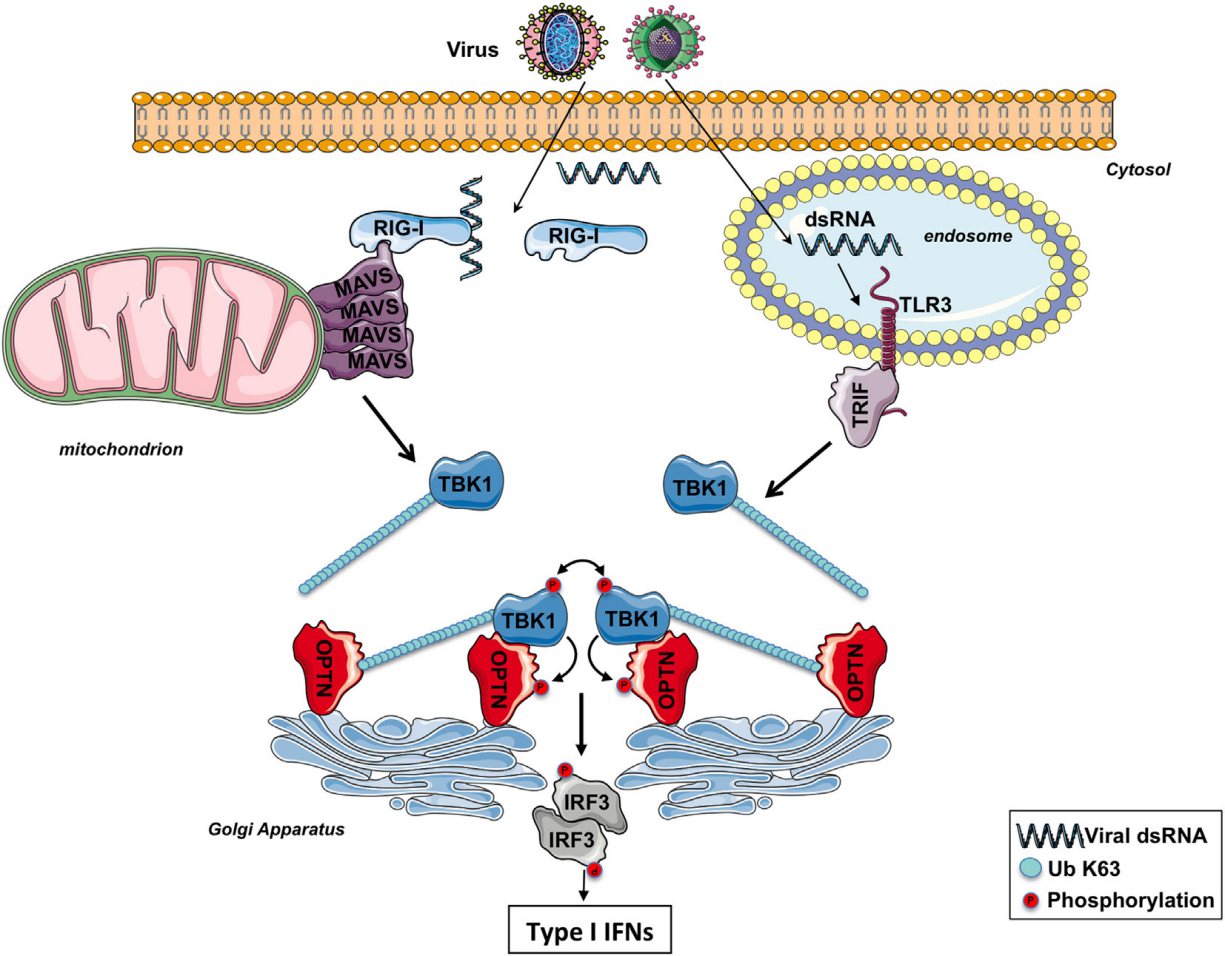
The involvement of optineurin (OPTN) in TBK1 activation following RIG-I-like receptors or toll-like receptor (TLR) 3 stimulation. After infection, viral double-strand RNAs (dsRNAs) are sensed by RIG-I in the cytosol or TLR3 in the endosomes. The corresponding adaptors, MAVS and TRIF, trigger the formation of specific signalosomes leading to the ubiquitination of TBK1. At the Golgi apparatus, OPTN recruits ubiquitinated TBK1 *via* its ubiquitin-binding domain, leading to the *trans*-autophosphorylation of this kinase. Activated TBK1 then phosphorylates IRF3, leading to the production of type I interferons (IFNs). TBK1 may directly interact with the N-terminus part of OPTN and promotes its phosphorylation. The function of the phosphorylation of TBK1 remains to be determined.

It has been proposed that the binding of the K63-linked poly-ubiquitin chains anchored on TBK1 to the UBD of OPTN likely promotes the oligomerization of TBK1–OPTN complexes, initiating the *trans*-autophosphorylation and TBK1 activation (39) (Figure 2). Because of its ability to bind to poly-ubiquitin chains through its UBD, OPTN functions therefore as a positive regulator in the activation of TBK1 (59, 60). Nevertheless, a recent study has reported that the TBK1-interacting N-terminus of OPTN is also required for TBK1 activation as cells isolated from mice expressing a mutant of OPTN without the N-terminus domain of OPTN (OPTNΔ157) have a reduced TBK1 activation, no phosphorylation of OPTN Ser(187), and the IFNβ responses is significantly decreased (61). The role of the phosphorylation of Ser(187) (Ser177 in human) of OPTN in the context of innate antiviral immunity remains to be determined. During mitophagy, it has been reported that TBK1 phosphorylates OPTN on Ser473 and 513 to increase its ability to bind polyubiquitin chains (49). It would be interesting to explore whether TBK1 also phosphorylates OPTN on these serine residues after TLR3 or RLR stimulation to amplify the sensing of poly-ubiquitinated TBK1.

Optineurin silencing with multiple siRNAs in different cell lines, OPTN knockout with CRISPR/Cas9 technology and the use of primary cells as MEFs or BMDMs from OPTN-deficient mice have clearly validated the crucial role of this adaptor in TBK1 activation after TLR3 or RLRs stimulation (44, 62). Moreover, a study has reported that osteoclast precursors from OPTN-mutant mice generate peculiarly low levels of IFNβ in response to RANKL (63). OPTN seems to also play a crucial role in TBK1 activation after TLR4 stimulation (59–62). Finally, it has been recently reported that OPTN, again through its poly-ubiquitin binding activity, is required for TBK1 activation consequently to mitochondrial depolarization confirming the involvement of OPTN, in the activation of this kinase (49, 50). Nevertheless, OPTN was initially reported to negatively regulate the induction of IFNβ in response to RNA virus infection based in part, to the silencing of OPTN in HEK293 cells with siRNAs (64). Another study has also used several siRNAs to knock down OPTN in HEK293, HeLa, or MEFs, but the authors have obtained the opposite result (44). This discrepancy is hard to explain but the use of OPTN-mutant- or -deficient mice have finally confirmed the role of OPTN as an important factor for the production of IFNβ through TBK1 activation (44, 60–62). A more recent work has also suggested that OPTN negatively regulates the IFN response in a cell cycle-dependent manner (65). Most of the conclusions of this study are based on the use of only one single clone of HeLa cells stably expressing an shRNA targeting OPTN so that, to explain the discrepancy with the current dogma, this clone may respond strangely and differently after a stimulation. Moreover, if OPTN does control the IFN production in a cell cycle-dependent manner, how may this protein then function in quiescent or non-dividing cells that are also infected by viruses?

Viruses have developed and selected a series of different stratagems to overcome the highly sophisticated mechanisms of defense of the hosts that they infect. During the course of pathogen–host co-evolution, some viruses have therefore acquired the capacity to impede the innate immune response by targeting and neutralizing host proteins (66). Interestingly, the observation that the NS3 protein of the Bluetongue virus binds and neutralizes OPTN at the Golgi apparatus, thus diminishing TBK1 activation and the ensuing IRF3 signaling (44), confirms the point that OPTN is required for activation of this kinase. Hence, this RNA virus, as a strategy to inhibit the innate immune response to favor its replication after infection, thwarts TBK1 activation by acting on OPTN. Likewise, other viruses have developed similar approaches for targeting and neutralizing other adaptor proteins implicated in TBK1 activation. For instance, the Gn protein of hantaviruses prevents the creation of TBK1 complexes where the kinase auto-activates, so impeding the downstream signaling (67). Furthermore, the vaccinia virus protein C6 targets other TBK1 adaptor proteins as TANK, NAP1, and SINTBAD, to inhibit the *trans*-autophosphorylation of the kinase and the resulting activation of the transcription factors IRF3 and IRF7 (68).

In the context of innate immunity, OPTN not only functions as a sensor at the Golgi apparatus for ubiquitinated TBK1 to promote its *trans*-autophosphorylation. Indeed, OPTN has also been described to be a substrate of TBK1. TBK1 actually phosphorylates OPTN on the Ser177 residue and this post translational modification strengthens the association between OPTN and LC3 (ubiquitin-like microtubule-associated protein light chain 3, a protein that decorates autophagosomal membranes) during xenophagy for the eradication of cytosolic *Salmonella* and to counteract the proliferation of the intracellular bacteria (69). It has been demonstrated by two independent studies that the RNA polymerase III senses viral or bacterial DNA in the cytosol and through the RLR signaling pathway triggers the production of type I IFNs (70, 71). So a bacterial infection can promote TBK1 activation at the Golgi apparatus through OPTN after stimulation of the RLR signaling pathway. Thus, in one hand, active TBK1 initiates the production of type I IFNs, and on the other hand, OPTN is phosphorylated by p-TBK1^S172^ to clear the invading bacteria by xenophagy.

An interaction with Rab8 allows the association of OPTN to the Golgi apparatus, and the interacting domain of OPTN is localized between amino acids 141–209 (72). This domain encompasses therefore Ser177 so that the phosphorylation of this serine residue after TBK1 activation (61, 69) may disrupt the binding of OPTN to Rab8. Consequently, the formed TBK1–OPTN complexes (61) are free to leave the Golgi membranes to phosphorylate TRIF at the endosomes or MAVS at the mitochondria for the recruitment of IRF3 and its phosphorylation (51). It may explain why in some models, p-TBK1 is actually detected at the mitochondria (52, 53). While TBK1 initially *trans*-autophosphorylates at the Golgi apparatus after RLR activation (44), the description that p-TBK1^S172^ accumulates later at the centrosome (44) reinforces the idea that active TBK1 (likely in complex with OPTN) is released from the Golgi membranes to phosphorylate its substrates.

Some studies have reported that other adapters, such as NAP1 or SINTBAD, are involved in TBK1 activation during antiviral responses (16, 73). Interestingly, like OPTN, NAP1 and SINTBAD are partially localized at the Golgi apparatus (44) confirming the crucial role of this organelle in innate immunity. Further investigations are needed to clarify if these adapters work together with the Golgi apparatus to promote activation of TBK1 and if so, by which mechanisms. Indeed, the observation of a low production of IFN-β but nevertheless present in the cells deficient for OPTN suggests that factors other than OPTN contribute to the activity of TBK1 or that this partial response depends on the activity of IKKε, which unlike TBK1 does not interact with OPTN (59, 74). It would be interesting to establish the relative proportion of the different complexes of TBK1, if they are activated selectively by different ligands and if there can be a synergistic effect between them.

Optineurin at the Golgi apparatus senses ubiquitinated TBK1 next allowing its activation through *trans*-autophosphorylation (44). Further studies are required to explore whether the Golgi by itself provides a suitable milieu for TBK1 activation (for instance, does the targeting of OPTN to other membranes impair TBK1 activation?) or whether this organelle only functions as a platform where TBK1 signalosomes form and mature. Finally, a crystal structure of ubiquitinated TBK1 in complex with OPTN would be very useful to fully understand the mechanisms by which TBK1 is activated and therefore how it might be modulated for therapeutic purpose.

## Concluding Remarks

After RNA virus infection, recent findings suggest that OPTN, at the Golgi apparatus, senses and recruits, through its UBD, K63-linked poly-ubiquitinated TBK1 leading to the *trans*-autophosphorylation of this kinase and ensuing IRF3 phosphorylation promoting the production of type I IFNs (44). Moreover, the description that RNF121, an E3 ubiquitin ligase anchored into the Golgi apparatus, is required for an efficient signaling conducting to NF-κB activation (75) suggests that while this organelle resides at the intersection of the secretory, lysosomal, and endocytic pathways, it also acts as a core required for the formation and/or maturation of complexes that promote the activation of the IRF3 and NF-κB signaling pathways. Whether the Golgi apparatus serves as a relay for other signaling pathways remain to be determined and deserve to be explored.

## Author Contributions

Each author has participated in the writing of this review.

## Conflict of Interest Statement

The authors declare that the research was conducted in the absence of any commercial or financial relationships that could be construed as a potential conflict of interest.
